# Elements of Health and Principles of Female Hygiene

**Published:** 1853-05

**Authors:** 


*Elements of Health and Principles of Female Hygiene.* By E. J. TILT, M.D. Senior Physician to the Farringdon General Dispensary and Lying-in Charity, &c. Philadelphia: Lindsay & Blackiston, 1853.

IN the preface the author says:—"As there exists no work on this subject, the present volume is intended to supply the *desider-*

*atum*, and we trust, that, notwithstanding deficiencies, it may be found acceptable."

The book is written in a popular style, and treats of the management and diseases of the female from birth to the age of eighty years. That portion of it devoted to infancy and childhood, we think should be read by every mother. Although designed for non-professional readers, it is scientific in its character, and may prove, as it seems to us, an antidote to many of the medical delusions of the day. J.

